# The *HER*-2 as a Target Gene of Curcumin to Protect Hepatocytes Against the Arsenic-induced Carcinoma in Mice

**Published:** 2017-04-01

**Authors:** Mahdi Ahadi, Vahid Naseh, Masoud Salehipour

**Affiliations:** 1 *Dept. of Biology, Faculty of Biological Sciences, Parand Branch, Islamic Azad University, Parand, Iran*

**Keywords:** *HER-2*, Hepatocellular Carcinoma, Curcumin, Arsenic

## Abstract

**Background & objective:**

The *HER-2 *gene is an important on co protein overexpressed in many types of cancers. The current study hypothesized that curcumin downregulates *HER-2* and inhibits the signal transduction pathway of PI3K/Akt, MAPK, and activation of NFκB, which could be useful to treat overexpressed-HER-2 hepatocellular carcinoma (HCC).

**Methods:**

In the current study, 40 male NMRI (Naval Medical Research Institute) mice were divided into 4 groups of 10 as follow: Group1 (control group) only received 5 mL/kg corn oil, group 2 (poisoned group) received 30 mg/L arsenic (As_2_O_3_) dissolved in water, group3 (curcumin treated), and group 4 (curcumin and arsenic treated) received 10 to 20mg/5mL/kg for 60 days. Once experimental period was completed, liver samples were collected. The analysis of the gene expression was performed by real-time polymerase chain reaction (PCR) technique.

**Results:**

Gene expression analysis showed that curcumin had significantly downregulated the activity of *HER-2*, in poisoned mice.

**Conclusion:**

According to the current study results, it could be concluded that curcumin has the inhibitory potential toward HER-2-overexpressed HCC.

## Introduction

Hepatocellular carcinoma (HCC) is defined as a primary liver malignancy, which principally occurs in patients affected by the chronic hepatic disease and cirrhosis (1, 2). Tumors include local expansion, intra hepatic spread, and distant metastases (2). In addition, arsenic in the environment is etiologically associated with tumor development in a variety of tissue, including skin, bladder, lung, liver, and prostate gland (3, 4). The most important source of environmental arsenic exposure in most populations is drinking water, in which inorganic forms of arsenic (trivalent arsenite and pentavalent arsenate) are predominant (5). Curcumin (diferuloylmethane) is naturally a yellow pigment obtained from the rhizome of the plant *Curcuma* *longa* L. The powdered rhizome of this plant, called turmeric, is usually utilized to prepare curries. Curcumin, as a polyphenol with a diarylheptanoid structure containing α, β-unsaturated ketones, is considered as the major active part of turmeric (6, 7). In addition to its broad spectrum of pharmacological activities, the anticancer properties of curcumin (8, 9), and its capability to downregulate *epidermal growth factor receptor* *(EGFR)* and *HER-2 *oncoproteins (9, 10) are reported in many studies. Also, curcumin can have an impact on several pathways such as PI3K/Akt and mitogen-activated protein kinases (MAPK) (11, 12). Additionally, curcumin is a potent anti-inflammatory compound (13). Recently, several studies indicated that the deregulated inflammatory pathways play a pivotal role in a large number of chronic diseases, including cancer (14). The leading mechanism by which curcumin serves as an anti-chronic inflammatory compound, and drives cancer initiation and progression is via increasing production of pro-inflammatory mediators such as cytokines, chemokines, reactive oxygen species (ROS), overexpression of cyclooxygenase (COX-2), matrix metalloproteinase (MMPs), oncogenes, intracellular signaling pathway mediators, transcription factors including signal transducer and activator of transcription 3 (STAT3), nuclear factor κB (NF-κB), activator protein 1 (AP1), and protein kinase B (AKT), that drive tumor cell proliferation, transformation, invasion, metastasis, and angiogenesis (15). Regarding the possible properties provided for curcumin in this introductory comment, it can be assumed as a beneficial agent in the treatment of *HER-2* positive cancers (16). Thus, the current study aimed at exploring the potential application(s) of curcumin on the *HER-2* expression in arsenic-poisoned mice.

## Material and Methods

**Induction of HCC and curcumin therapy**

In the current study, 40 male NMRI (Naval Medical Research Institute) mice weighing 30 to 35 gr were recruited. After being matched according to the body weight, the mice were divided into 4 different groups of 10: Group 1, control mice received 5 mL/kg/day corn oil (Sigma-Aldrich) for 60 days; group 2, (poisoned mice) received 5 mL/kg/day corn oil and 30mg/L arsenic (As_3_O_2_) for 60 days; group 3, treated mice received 5 mL/kg/day corn oil and 10 to 20 mg/5 mL/kg/day curcumin (Sigma-Aldrich) for 60 days; group 4, (treated mice) received 5 mL/kg corn oil and 10 to 20 mg/5 mL/kg/day curcumin and arsenic ш for 60 days. Also, 5 mL of the above mentioned components were given to the animals via gavage syringe. Subsequently, mice were housed in the cages with free access to water and standard food. All of the animal handling processes were performed according to the guidelines of Iranian Animal Ethics Society, School of Science, Islamic Azad University, Parand Branch. At the end of the 60-day treatments, liver samples were collected, and the expression of *HER-2* was analyzed by real-time polymerase chain reaction (PCR). 

**RNA isolation**

To isolate RNA from tissue, mice were sacrificed; the liver removed under aseptic situations and immediately frozen in liquid nitrogen. Liver tissue samples were homogenized in TRIZOL™ reagent (Sigma-Aldrich) using Mixer 301, and subjected to RNA extraction. The purity extracted RNA was determined by electrophoresis on an ethidium bromide pretreated agarose gel along with measuring absorption at 260/280 nm using the spectrophotometric method.

**Synthesis of cDNA and quantitative real-time PCR**

Five micrograms of RNA were reversely transcribed using reverse transcriptase enzyme for 1 hour at 37°C to synthesize cDNA. Quantitative changes of mRNA were assessed by quantitative real-time PCR (Bioneer, Exicycler™ 96 Korea) using SYBR green detection system contained SYBR green PCR Master Mix (Thermo Scientific). The sequences of the used primer are demonstrated in Table 1. The *β-actin* was used as a housekeeping gene, and each sample was normalized on the basis of its *β-actin* content. The mRNA encoding target genes were analyzed by employing real-time PCR method and were normalized by *β-actin* mRNA (as the housekeeping gene), using the 2-ΔΔCt formula.

**Histological staining of tissue**

Liver tissues were collected and fixed in 10% formalin after sacrifice. They were cut transversely or longitudinally to obtain ventricular sections or 4-chamber cross sections, respectively; then, embedded in paraffin and cut into 4-μm thick slices for morphological and pathological evaluations. Tissue sections were stained with hematoxylin and eosin (H&E), examined using a light microscope equipped with a charge coupled device (CCD) camera (KE2500, Nikon, Tokyo, Japan), and diagnosed by a clinical pathologist.

**Statistical Analysis**

Results were presented as mean± standard deviation (SD). The results were statistically analyzed using a one-way analysis of variance (ANOVA), followed by Tukey multiple comparisons for all tests by SPSS software version 18 (SPSS Inc., Chicago, IL, USA). P ≤0.05 was considered statistically significant.

## Results

**Fig 1:**
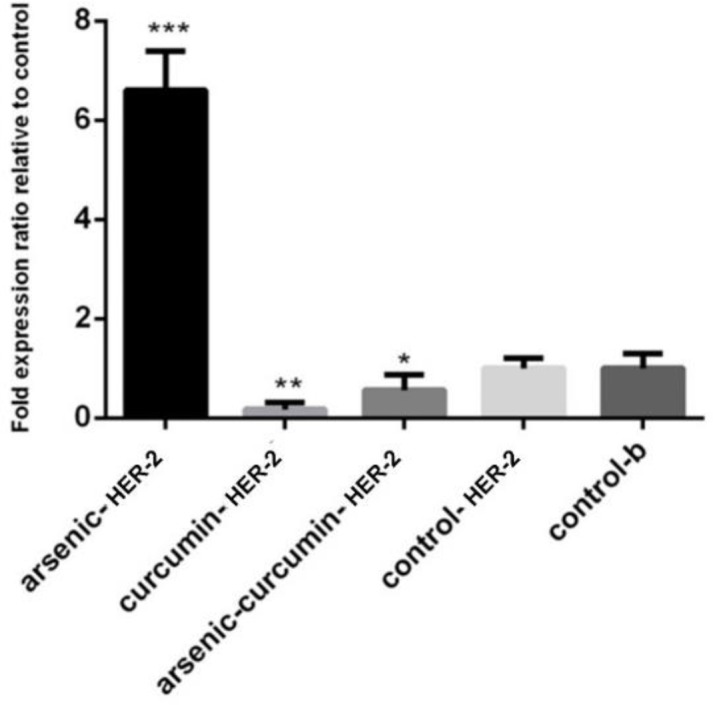
Expression of *HER-2 *at mRNA level in 4 groups: Group 1 (the control group) received corn oil, group 2 (poisoned group) received arsenic, group 3 (curcumin treated), and group 4 (curcumin and arsenic treated). Values are expressed as mean±SEM for all experiments. [*P <0.05 versus relevant control and housekeeping gene]

The current study enrolled 40 mice, and HCC was induced in them by arsenic ш. The mRNA levels of *HER-2* in the liver of normal control group were regarded as 100% expression, and the expression in the other groups were calculated accordingly. The current study results showed that the expression of *HER-2* was 6.6±057 in group 2, ( received only arsenic, P<0.001), 0.18±0.23 in group 3 (curcumin treated, P<0.01), and 0.56±0.13 in group 4 (curcumin and arsenic treated, P<0.05), while expression of *HER-2* in the control and *β-actin* control groups were 1.06±0.94 and 1.09±0.94, respectively (Figure 1).

**Fig 2:**
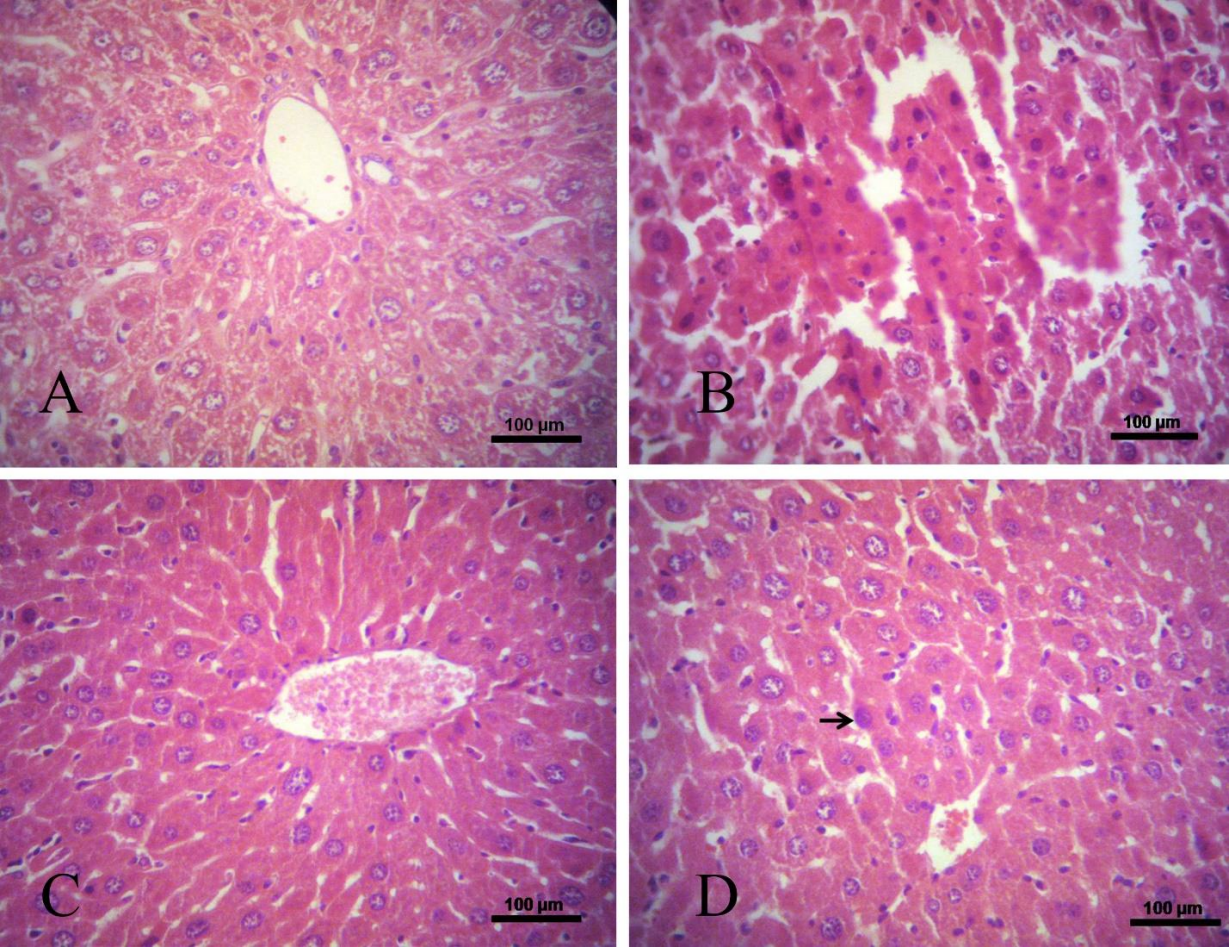
Photomicrographs of liver sections stained with hematoxylin and eosin (bar, 100 μm). (A) Control mice, showing normal hepatocytes architecture; (B) Liver of HCC mice treated with arsenic, showing HCC development; (C) Liver of HCC mice treated with curcumin, shows approximately normal architecture; (D) liver of HCC mice received arsenic and curcumin, pointer shows the helpful effect of curcumin on cancerous cells that are returning to normal state.

Results of the current study showed that curcumin effectively downregulated the *HER-2* expression in comparison with curcumin and arsenic treated group. Sections of liver tissue were stained with H&E illustrated that livers of the mice treated with arsenic developed HCC, and treatment of this group with curcumin showed that curcumin alone had the property to cure HCC. Also in the group that received arsenic and curcumin, results suggested that curcumin had beneficial effects to return the cancer cells back to normal status (Figure 2).

## Discussion

Primary liver cancer, principally hepatocellular carcinoma (HCC), remains the 8th largest cause of cancer in females and the 5th largest in males (17, 18). HCC is the 3rd cause of cancer-related deaths worldwide (19, 20). Also, HCC is a malignancy with acutely ominous prospects and a 5-year survival rate reported below 9% (21). In addition, the role of *HER-2 *was discussed in different studies. Similar to *HER-1*, the *HER-2* molecule is a member of a larger family of receptor tyrosine kinase overexpressed in various human malignancies such as liver, breast, ovarian, endometrial, cervical, lung, stomach, colon, mesenchymal, esophagus, bladder, and kidney cancers (22-26).

As a result, curcumin is an intelligent compound suppressing the expression of *HER-2* and might be a useful option for cancer therapy. The current study showed that curcumin can be considered as an effective agent that downregulates the *HER-2* expression in HCC. Consistent with the current study results, Hung et al., showed that the expression of *HER-2* significantly increased in HCC (27). Chan et al., found that turmeric (*Curcuma longa*) inhibits the secretion of tumor necrosis factor (TNFα) (28). In several other studies, the antioxidant, antitoxic, anti-inflammatory, and anticancer properties of curcumin are reported (29). Curcumin has powerful antioxidant effects to neutralize free radicals and the ability to collect ROS (30). Cao et al., reported that curcumin inhibited the growth of atypical glandular cells of undetermined significance (AGS cells) and induced apoptosis through the activation of Ras/ERK signaling pathway. They suggested that curcumin can be a potential agent to treat gastric carcinoma (31). 

Hu et al., reported that curcumin, with antioxidant and anti-inflammatory effects, can be helpful in cancer therapy (32). In agreement with the results of the current study, Wen Lai et al., demonstrated that curcumin could decrease the expression of* HER-2* in patients with breast cancer (33). Soo Chun et al., showed that curcumin prevented tumor progression via inhibition of ERK1/2, NFKB, and COX-2 (34). In parallel with the results of the current study, Reddy et al., showed that curcumin can reduce the progression of colon cancer via reducing the expression of *EGFR*, *ErbB-2*, *ErbB-3*, and / or *insulin-like growth factor 1 receptor (IGF-1R)* genes (35). Several scientific reports stated the carcinogenic effect of arsenic and its impact on *HER-2*. For instance, Chuan shu et al., reported that arsenic played a fundamental role to activate signaling mediators including miR-199/148 / ERBB2 / PKM2 / NF-kB to increase the expression of *hypoxia-inducible factor 1 (HIF-1), interleukin (IL)-8*, and finally increased angiogenesis and contributed to the processes of tumor progression and development (36). Simeonova et al., showed that arsenic can affect the activation of the MAPK/ERK pathway group of molecules, including nuclear transcription factor *AP-1, c-fos, c-jun, *and* c-myc* (37). Flora et al., claimed that arsenic can increase the production of ROS. ROS itself causes altered signaling pathway and regulates the expression of transcription factors. His study also pointed to the role of arsenic in the activation of MAPK/ERK pathway cascade through the activation of signaling and phosphorylation of *EGFR/MEK, EGFR/Ras/MEK, Src/EGFR* performed (38). Findings of the current study indicated that arsenic could lead to increased expression of *HER-2* and other oncoproteins; and curcumin, as a chemopreventive and therapeutic agent in HCC, has helpful properties including antioxidant and anti-inflammatory impacts, and the capability to regulate a variety of signaling mechanisms. In vitro and in vivo preclinical models indicate that curcumin, along with different curcuminoids, are potential curative agents for HCC. 

To draw a conclusion, considering the results of the current study, it could be suggested that curcumin has the complimentary potency to be developed as an antitumor operant to treat HCC. Additionally, maybe curcumin suppresses these types of hepatic tumors by balancing (up/down-regulating) the expression of *HER-2* in arsenic-poisoned mice. Maybe enrichment in other antioxidant and antitumor agents makes curcumin a possible compound to protect hepatic cells from arsenic-poisoned damage.
